# Deep brain stimulation for the treatment of substance use disorders: a promising approach requiring caution

**DOI:** 10.3389/fpsyt.2024.1435109

**Published:** 2024-07-12

**Authors:** Joseph T. Sakai, Jody Tanabe, Sharonya Battula, Morgan Zipperly, Susan K. Mikulich-Gilbertson, Drew S. Kern, John A. Thompson, Steven Ojemann, Kristen Raymond, Pamela David Gerecht, Katrina Foster, Aviva Abosch

**Affiliations:** ^1^ Department of Psychiatry, University of Colorado School of Medicine, Aurora, CO, United States; ^2^ Department of Radiology, University of Colorado School of Medicine, Aurora, CO, United States; ^3^ Department of Neurology, University of Colorado School of Medicine, Aurora, CO, United States; ^4^ Department of Neurosurgery, University of Colorado School of Medicine, Aurora, CO, United States; ^5^ National Institute on Drug Abuse, Bethesda, MD, United States; ^6^ Department of Neurosurgery, University of Nebraska Medical Center, Omaha, NE, United States

**Keywords:** substance use disorder, addiction, brain stimulation, neuromodulation, invasive, craving

## Abstract

Substance use disorders are prevalent, causing extensive morbidity and mortality worldwide. Evidence-based treatments are of low to moderate effect size. Growth in the neurobiological understanding of addiction (e.g., craving) along with technological advancements in neuromodulation have enabled an evaluation of neurosurgical treatments for substance use disorders. Deep brain stimulation (DBS) involves surgical implantation of leads into brain targets and subcutaneous tunneling to connect the leads to a programmable implanted pulse generator (IPG) under the skin of the chest. DBS allows direct testing of neurobiologically-guided hypotheses regarding the etiology of substance use disorders in service of developing more effective treatments. Early studies, although with multiple limitations, have been promising. Still the authors express caution regarding implementation of DBS studies in this population and emphasize the importance of safeguards to ensure patient safety and meaningful study results. In this perspectives article, we review lessons learned through the years of planning an ongoing trial of DBS for methamphetamine use disorder.

## Introduction

Substance use disorders (SUD) are common [e.g., 5-20% prevalence for alcohol use disorder alone (1)], and when severe, are often chronic, life-threatening disorders (2). Several evidence-based treatments for SUD are available but many patients do not respond to these interventions and new approaches are needed. To date most work has focused on psychosocial and pharmacological treatments. In recent decades, our understanding of the neurobiology of SUD has grown (3–6) and neuromodulation methods have been developed and tested for other disorders, paving the way for testing neuromodulation for SUD.

**Figure 1:**
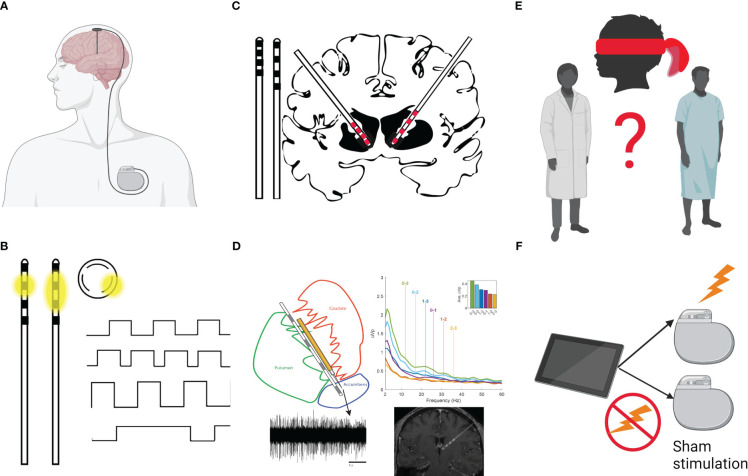
**(A)** The DBS system is composed of the lead (with electrodes at the tip), extension wires connecting the lead and Implantable Pulse Generator (IPG, battery). The neurosurgical procedure involves stereotactic placement of an electrode (lead) into specific brain targets. The lead is connected via an extension cable tunneled under the scalp, underneath the skin of the neck, and connected to a programmable battery (IPG) that is positioned underneath the skin of the chest and delivers electrical stimulation to one or more of the electrode contacts. **(B)** The tip of the lead generally has several electrodes (4 black squares here) spaced evenly apart. The battery can be programmed to send the stimulation through one contact (monopolar setting; e.g., with the case as the anode/+ and the electrode as the cathode/-) and the current will flow in a donut shape (dissipating with distance from the contact; see first inset image of a lead). Alternatively, the programmer can set one contact to serve as anode and one other as the cathode (bipolar setting, sending the current between contacts in an oval shape; see second inset lead image). Some leads allow “directional” stimulation (see circular inset image, which represents a cross-section of the lead). These leads allow sending the stimulation in one of 3 directions (e.g., anterior in a direction perpendicular to the lead). The shape of stimulation can help target specific structures based on patient imaging and offers alternative stimulation setting to mitigate reported side effects. The programmer can also control characteristics of the stimulation delivered. Programming includes designation of active contact(s), amplitude (voltage/mA), pulse width (µs), and frequency (Hz) of stimulation. The top image shows a stimulation setting with a rectangular waveform. The second wave form shows an increased frequency stimulation setting (more pulses/rectangles per second). The third wave form shows an increased amplitude (higher mA as shown by taller rectangles) and the final wave form shows a very long pulse width. **(C)** Leads vary in their spacing of the electrical contacts. To the left of the brain, two leads are shown side-by-side. The Medtronic leads B33005 (left; with 0.5mm spacing between contacts, which themselves are 1.5mm in length) and B33015 (right; with 1.5mm spacing between contacts). The brain image shows a typical approach to the Nucleus Accumbens (NAc) with the B33005 on the left and the B33015 on the right. Given an average height of the NAc here is estimated to be ~7mm (Neto et al., 2008), the B33015 lead placement (right part of the brain image) would allow the two most distal contacts to be placed in the NAc and electrode coverage superiorly into the anterior limb of the internal capsule. B33005 lead placement (left side of the brain image) in the NAc allows coverage similar to the first 3 contacts of the B33015 (e.g., bullet or rounded tip of the lead plus three 1.5mm contacts and two 1.5mm inter-contact spaces = bullet plus four 1.5mm contacts and three 0.5mm intercontact spaces = bullet + 7.5mm). Thus, the B33005 may allow tighter control to stimulate different portions of the NAc, but does not allow full coverage of the Anterior Limb of the Internal Capsule. **(D)** The Medtronic Sensight™ leads used in the authors’ trial, allow “sensing” or recording of local field potentials (LFP) or electrical potentials in the extracellular space (e.g., from a cluster of neurons in the nearby region). Unlike EEG, which has excellent temporal resolution but poor spatial resolution and MRI, which has excellent spatial resolution but limited temporal resolution, LFP measurement allows recording activity directly from a brain structure (in the authors’ study the NAc/BNST/ALIC; local field potential and MRI data represent modified versions of data published in Duffy et al., 2023) in real time (e.g., while presenting a cue craving paradigm). Sensing cannot be conducted while using bipolar stimulation and stimulation must be set using only the middle contacts in a monopolar fashion while recording. **(E)** Blinding of the patient and of the study team represents a major methodological challenge in the design of DBS studies. Because of the nuances in programming for substance use treatment, the Addiction Psychiatrist is appropriately the de facto programmer. Although the patient is blinded to the treatment arm, the psychiatrist not only knows the assigned arm but also adjusts program settings based on subjective clinical responses. There are ways to mitigate this confound, for example, by standardizing rather than individualizing programming or having an unblinded study member reset programming according to treatment arm after the psychiatrists’ evaluation. Each approach will have different limitations. Unfortunately, we are unaware of programming tablets which would allow blinding of the individual conducting programming. **(F)** Placebo response rates are relatively high for psychiatric conditions. As is the case for any new intervention, a randomized controlled trial of DBS should be the gold standard. Open-label DBS trials provide information on safety, tolerability, time to response and optimization of stimulation parameter settings. Many initially promising open-label DBS studies for psychiatric disorders, however, have been followed by placebo-controlled trials, with disappointing results. Figure created with BioRender.com.

DBS is a neurosurgical procedure, which was first FDA-approved in the United States in 1997 for the treatment of tremor (7), and is well-established for treating movement disorders. Approximately 208,000 individuals have undergone DBS surgery worldwide (8). DBS involves stereotactic placement of an electrode (lead) into specific brain targets, which is connected via an extension cable to an implanted pulse generator (IPG). The IPG is positioned underneath the skin of the chest and is programmed by a treating clinician via a wirelessly-connected tablet to deliver electrical stimulation to one or more of the electrode contacts (**Figure 1**). DBS is an expensive procedure that carries surgical risk, and commonly requires at least an overnight hospital admission. Compared to transcranial magnetic stimulation (TMS), DBS provides targeted, constant, precise and highly titratable stimulation to deep brain structures.

The exact mechanism of DBS’ therapeutic effect is unknown, likely multifactorial, and may involve axonal stimulation, inhibition, and disruption of pathologic oscillations. Current research is aimed at understanding the effects of DBS at the molecular, cellular, and systems levels (9), determining optimal targets within neural networks and optimal stimulation parameters. Early animal work has suggested that DBS of the ventral tegmental area may attenuate drug-induced dopamine increases in the nucleus accumbens (NAc) following intravenous drug administration (10), and DBS of the NAc shell may reduce the extinction period for conditioned place preference and prevent drug-primed reinstatement (11). Early work in humans focusing on DBS and multiple SUDs has been promising as reviewed previously (12), suggesting high remission rates. In the only double-blind randomized controlled trial (RCT) published to date, a reduction in alcohol use was observed, but the trial failed to meet its primary endpoint of abstinence (13). Unlike DBS for the treatment of movement disorders, which has a well-characterized and acceptable risk-benefit profile, DBS for SUD is off-label, and trials require careful planning to address multiple population-specific concerns.

### Safety and patient selection

DBS surgery is generally considered to be low risk, with a permanent surgical morbidity rate of ~1% (14). The most serious risks include intracranial hemorrhage, stroke, and infection sometimes requiring explantation of some or all DBS components. It is currently unclear whether associated morbidity and mortality differs in persons with SUD compared with other populations but available data to date do not suggest substantive differences (**Table 1**). Still, as more patients with SUD undergo surgery and different neurosurgical targets are tested/adopted, these results may change.

**Table 1:** Adverse events (AEs) and serious adverse events (SAEs) reported for deep brain stimulation for substance use disorder.

Adverse events reported	Substance Use Disorder & Sample Size	Study Design	Outcome Measures	Reference
**Surgery-related:** 6 AEs related to surgery **Post-Operative:** (1) Battery depletion (11 months) (2) Lack of drive, sleepiness (3) Device turned off (requested by participant) (4) Relapse (5) Decreased libido, erectile dysfunction (6) Depressive syndrome (7) Difficulty falling or staying asleep (8) Headache 38 adverse events noted during first 6 months Stim on first 6 months – > 24 AE, 7 SAE (n=4) Stim off first 6 months – > 14 AE, 7 SAE (n=2) 82 AEs in 12 month open label follow up (n=9) 69% of all AEs rated as mild or moderate; 86% of AEs in first 6 months were resolved, 89% of AEs in subsequent 12 months were resolved SAEs were mostly related to relapse and inpatient treatment No deaths, no lasting disabilities	Alcohol Use Disorder (n=12) *Participants all male	Randomized, double-blind, followed by open label 6 months on vs. 6 months off stimulation, followed by 12 months all-on stimulation	**Primary:** - Time to first alcohol use **Secondary:** - Mean proportion of abstinent days - Number of heavy drinking days - Mean alcohol consumption per day - Alcohol craving - Anhedonia - Depressive Symptoms - Anxiety - Quality of Life - Global Functioning	(Bach et al., 2023) (13)
**Surgery-related:** (1) Intracranial hemorrhage (<3ml) without neurologic deficit (n=1) (2) fever (n=1) (3) headache (n=1) **Stimulation-related** (reversible when stim turned off): (1) Dizziness (2) Agitation/irritability (3) Sweating (4) Difficulty falling asleep (first night on stim) (5) Self-reported memory decline with chronic stim	Opioid Use Disorder (n=8)	Open label, followed for 3+ years	**Primary:** - Duration of drug-free time after surgery **Secondary:** - Abstinence rates at 24 months - Severity of cravings - Quality of Life, as evaluated by Medical Outcomes Study 36-Item Short-Form Health Survey - Symptom Check List-90 - Yale-Brown Obsessive-Compulsive Scale - Hamilton Depression rating scale	(Chen et al., 2019) (15) *Ge and colleagues (16) *report on same patients per Mahoney* (12) *and does not include adverse events*
**Intraoperative:** No adverse events reported **Post-Operative:** (1) Infection (n=1 at 12 months originating a the IPG and spreading rostrally, requiring explantation) (2) Hypomania (n=1, resolved within 24 hours of reducing 4.5V – > 3.5V) (3) Scalp pruritis (n=1, prolonged, requiring steroid cream) (4) Acute depression (n=1 after inadvertent device deactivation, patient reported increased cravings, worsening mood and increased anxiety; resolved fully within 12 hours of reactivation) (5) Relapse to alcohol (n=6) (6) Fatigue (n=1, sleeping >10 hours per night) (7) Cosmetic concerns (n=1) (8) Headache (n=5, all resolved by 12 weeks) (9) Oropharyngeal carcinoma (n=1, found on post operative imaging) (10) Myocardial infarction (n=1, fatal 14 months postoperatively)	Alcohol Use Disorder (n=6)	Open label, followed for 12 months	**Primary:** - Safety - Changes in self-reported alcohol consumption 6 months after implantation compared to baseline, using the timeline follow-back **Secondary:** - Alcohol Use Disorder Identification Test - Obsessive-Compulsive Drinking Scale - Alcohol Urge Questionnaire - Alcohol Dependency Scale - Hamilton Depression Rating Scale - Beck Anxiety Inventory - Beck Depression Inventory - Liver Function Tests (AST, ALT)	(Davidson et al., 2022) (17)
**Post-Operative:** Patient A noted to have: (1) Insomnia (2) Hypomania (2 days, resolved with changes to stim) Patient B noted to have: (1) Right DBS lead misplacement (outside the NAc, near the globus pallidus) (2) Anxiety (with 4.5V, reduced to 3.7V) (3) Hypomania symptoms (with 4.5V) and 1 week of hypomania (with 3.7V, resolved with reduction to 3.3V) (4) Insomnia (5) Bruxism (resolved with reduction to 3.3V) (6) Recurrent depressive symptoms	Stimulant Use Disorder, Methamphetamine Type (n=2) *Both participants male	Open label, followed for 18 months (for patient A) and 30 months (for patient B)	**Primary:** - Duration of post-surgical drug- free period	(Ge et al., 2019) (18)
**Intraoperative:** (1) Metallic taste (with stimulation at contacts 0, 1) (2) Dysautonomic phenomena like hot flushes, sweating (with “high- voltage” stimulation at contacts 0) (3) Contralateral “hemi- smile” automatisms (with stimulation at contacts 1) **Post-Operative:** (1) Unpleasant warmness (contact 0 near hypothalamus, above 3V left and 4V right) (2) Sweating (contact 0 near hypothalamus, above 3V left and 4V right) (3) Flushing (contact 0 near hypothalamus, above 3V left and 4V right) (4) Metallic taste (occasional, with >3V contact 1) (5) Diminished libido (4-5V on contacts 0-1 bilaterally) (6) Weight gain (10kg “transient”) No severe or permanent adverse events	Stimulant Use Disorder, Cocaine Type; Opioid Use Disorder, In remission on Methadone (n=1)	Phase 1 (9 months) – assessment, surgery and stim parameter optimization Phase 2 (9 months) – double-blind crossover control 2 months on/off alternatively for 6 months, and 3 months single-blind off-stimulation Phase 3 (12 months) – open label stimulation	**Primary:** - Weeks free of drug consumption **Secondary:** - Percent negative urinalysis - Craving visual analog scale - Drug desire questionnaire - Yale-Brown Obsessive Compulsive Scale - Clinical Global Impressions, severity, improvement, and therapeutic efficacy	(Gonçalves-Ferreira et al., 2016) (19)
**Intraoperative:** No adverse events reported **Post-operative:** (1) Hypomania (n=1, resolved with changes to stimulation parameters)	Alcohol Use Disorder (n=3) *All participants male	Open label	**Primary:** - Alcohol craving - Abstinence from Alcohol	(Heinze et al., 2009) (20) *Mahoney* (12) *notes that Heinze* (20)*, Müller* (21)*, Müller* (22)*, and Voges* (23) *reported on the same pts, with Müller* (22) *and Voges* (23) *including 2 additional pts.*
**Intraoperative:** No adverse events reported **Post-Operative:** (1) Hypomania (resolved with changes to stimulation parameters)	Alcohol Use Disorder (n=1)	Off-label study protocol, PET scanning at 18 months at 22 months post-implantation	- Reward processing using a gambling paradigm - Win- and loss-related activations as measured by PET	(Heldmann et al., 2012) (24)
**Intraoperative:** No adverse events reported **Post-Operative:** No adverse events reported	Alcohol Use Disorder (n=1) *Patient with comorbid anxiety and depression, which was the target of DBS treatment	Open label, followed for 12 months	Incidental finding of decreased alcohol consumption, correlated with changes in WHO Alcohol Use Disorder Identification Test score, carbohydrate deficient transferrin value, and gamma-glutamyl transferase value	(Kuhn et al., 2007) (25)
**Intraoperative:** No adverse events reported **Post-Operative:** No adverse events reported	Tobacco Use Disorder (n=10) *Patients with comorbid Tourette’s syndrome, obsessive-compulsive disorders, or anxiety disorders, which were the targets of DBS treatment	Open label, retrospective chart review	**Primary:** - Successful cessation of smoking - Fagerstrom Test for Nicotine Dependence **Secondary:** - Changes in primary mental disorder	(Kuhn et al., 2009) (26) *Per Mahoney patient from Kuhn et al., 2007* (25) *is included in Kuhn et al., 2009* (26)
**Intraoperative:** No adverse events reported **Post-Operative:** (1) Elevated state anxiety only at time 4 during period with stimulation off	Alcohol Use Disorder (n=1)	Open label, with short (24 hr) wash out periods, followed for 12 months	**Primary:** - Alcohol craving (Alcohol Dependence Scale, AUDIT, Craving Believe Questionnaire, Inventory of Drinking Situation, Obsessive Compulsive Drinking Scale) - Alcohol use and dependence **Secondary:** - Error processing (error-related negativity) and anterior mid- cingulate cortex functioning	(Kuhn et al., 2011) (27)
**Intraoperative:** No adverse events reported **Post-Operative:** (1) Seizure 2 days post- operation (n=1, this patient was noted to have a history of epileptic seizures)	Opioid Use Disorder (n=2) *Participants with other co-occurring substance use disorders	Open label, followed for 12-24 months	**Primary:** - Craving, measured using visual analog scale - Abstinence from Heroin **Secondary:** - Quality of life, as measured by the Modular System for Quality of Life	(Kuhn et al., 2014) (28)
**Intraoperative:** No adverse events reported **Post-Operative:** No adverse events reported No serious adverse events	Opioid Use Disorder; Benzodiazepine Use Disorder (n=1)	Open label, 12 week endpoint and at 12 month extended follow up time point	**Primary:** - Craving, measured using a visual analog scale - Urine toxicology - Frontal/executive function and risk-taking behavior, measured using the balloon analog risk task - Glucose metabolism as measured by FDG-PET - Heart rate variability - Impulsivity, measured using the Barratt Impulsiveness Scale - Depression and anxiety, measured with the Comprehensive Psychopathological Rating Scale	(Mahoney et al., 2021) (29)
**Intraoperative:** No serious adverse events **Post-Operative:** (1) Weight gain (8 kg during the “first months” after DBS surgery – though note subsequently lost 44kg “without any effort” reaching her goal of 71kg.)	Nicotine Use Disorder (n=1) *Patient with comorbid OCD and obesity, with OCD as the target treatment	Open label, followed for 24 months	**Primary:** - OCD symptoms, measured with the Yale-Brown Obsessive- Compulsive Scale **Secondary:** - Anxiety, measured by the Hamilton Anxiety Scale - Depression, assessed with the Hamilton Depression Scale - Smoking Cessation and Craving, measured byFagerstrom Test for Nicotine Dependence) - Weight Loss/Gain	(Mantione et al., 2010) (30)
**Intraoperative:** No adverse events reported **Post-Operative:** (1) Hypomania (n=1, onset 2 weeks after surgery x 1 week, resolved with change in stimulation parameters)	Alcohol Use Disorder (n=3) *All patients were male	Open label, followed for 12+ months	- Symptom Check List 90 - Alcohol-related thoughts and drinking behavior, assessed with the Obsessive-Compulsive Drinking Scale - Craving, measured by the Alcohol Urge Questionnaire - Abstinence from alcohol, number of and duration of relapses	(Müller et al., 2009) (21)
**Intraoperative:** No adverse events reported **Post-Operative:** (1) Death (n=2; 1 patient died 8 years after initiation of DBS and another after 4 years of DBS treatment; no causal relationship between DBS and death was found or suspected) (2) Depressive episode (n=2; 1 patient with onset 36 months after start of DBS; second patient with onset 24 months after onset of DBS – both treated to remission with SSRI and psychotherapy) No “severe or long-standing side effects”	Alcohol Use Disorder (n=5) *All participants were male	Open label, followed for multiple years	- Symptom Check List 90 - Alcohol-related thoughts and drinking behavior, assessed with the Obsessive-Compulsive Drinking Scale - Craving, measured by the Alcohol Urge Questionnaire - Abstinence from alcohol, number of and duration of relapses	(Müller et al., 2016) (22) Müller (21) includes the 3 patients from Müller (22) with 2 additional participants
No serious adverse events; no adverse events related to device, surgical procedure, or stimulation	Opioid use disorder (n=4) *All participants were male, with other co-occurring substance use disorders **One participant discontinued his enrollment, and the device with explanted 11 weeks post-implantation	Open label, followed for 12 months	**Primary:** - Safety and tolerability - Opioid and other substance use, measured by qualitative and quantitative urine toxicology **Secondary:** - Substance craving, measured by visual analog scale - Emotional symptoms, measured by the Comprehensive Psychopathological Rating Scale - Glucose metabolism as measured by FDG-PET neuroimaging	(Rezai et al., 2023) (31)
**Intraoperative:** No adverse events reported **Post-Operative:** (1) Stimulation of middle contacts led to increased craving and drug use	Opioid Use Disorder (n=1)	Open label, followed for 6+ months	- Average daily heroin use - Intention/desire to use heroin, measured by the “desire and intention” scale of the desires for drugs questionnaire	(Valencia-Alfonso et al., 2012) (32)
**Intraoperative:** No adverse events reported **Post-Operative:** (1) Seizure (n=1, generalized seizure approximately 3.7 years after surgery, imaging showed electrode displacement and both electrodes were subsequently replaced) (2) Hypomania (n=1, resolved by switching from bipolar stimulation to monopolar stimulation at the distal contact, and reducing stimulation energy) (3) IPG replacement (n=4) (4) “Inner restlessness” (n=1, noted when the battery was low)	Alcohol Use Disorder (n=5)	Open label, followed for 32-48 months	- Duration of abstinence - Number of relapses - Obsessive-Compulsive Drinking Scale - Alcohol Urge Questionnaire - Rey Auditory Verbal Learning Test - Hamburg Wechsler Intelligent Test for adults - Subtest 3 of the Leistungspruefsystem - Multiple Choice Word Test-B - Trail Making Test A & B - Symptom Checklist 90	(Voges et al., 2013) (23) Voges (23) includes 2 of the participants from Müller (21)
**Intraoperative:** No adverse events reported **Post-Operative:** (1) Noted to have adjusted stimulation parameters in the first month to “avoid agitation and hypomania, which can be a side effect of DBS treatment.” It is unclear if the patient experienced this.	Stimulant Use Disorder, Methamphetamine Type (n=1)	Open label, followed for 12 months	- Abstinence, assessed by self-report, urine and hair drug testing - Striatal dopamine transporter (DAT) levels, measured by 11C-CFT PET imaging - Craving, assessed with a Visual Analogue Scale - Obsessive-Compulsive Drug Use Scale -	(Zhang et al., 2019) (33)
**Peri-Operative:** (1) Mild confusion (2) Urinary incontinence **Post-Operative:** No adverse events reported	Opioid Use Disorder (n=1)	Open label, followed for 72 months, though system was removed after 3 years at request of patient and family	**Primary:** - Abstinence from heroin use **Secondary:** - Degree of tobacco use - Memory and IQ, using Wechsler Memory Scale Memory Quotient score and IQ test - Minnesota Multiphasic Personality Inventory - Symptom Checklist 90 - Depressive symptoms, assessed via a self-rating depression scale - Anxiety, measured via a self-rating anxiety scale	(Zhou et al., 2011) (34)
**Intraoperative:** No adverse events reported **Post-Operative:** (1) 19 kg weight gain at 6 month follow-up (but pt was able to lose 10kg in the subsequent 6 months working with endocrinologist)	Multiple co-occurring substance use disorders (n=1) *Pt had developed addictions to bucinnazine, morphine, zopiclone, alprazolam	Open label (DBS plus anterior capsulotomy), followed for 12 months	**Primary:** - Drug craving, using the Visual Analogue Scale - Drug abstinence **Secondary:** - Depressive symptoms, using the Hamilton Depression rating scale, Beck Depression Inventory - Anxiety, measured with the Hamilton Anxiety Rating scale, Beck Anxiety Inventory - Manic symptoms, via the Young Mania Rating scale - Presence of adverse effects, assessed with the Monitoring of Side Effects Scale - Sleep quality, via Pittsburgh Sleep Quality Index - 36-Item Short Form Health Survey - Work and Social Assessment Scale - Cognitive Function, measured via COG-STATE	(Zhu et al., 2020) (35)

Prior work has reported on adverse events for neurological conditions (36) and for obsessive compulsive disorder (37). Types of adverse events for DBS of SUD mirror many of those seen in OCD. However, rates in SUD populations are difficult to determine due to differential approaches to measurement of adverse events and lack of information provided in some studies.

Which patients? Unlike medication trials that often recruit broadly, studies of DBS generally have been reserved for patients with severe, long-term, treatment-refractory SUD (12). Patient selection criteria have been well described for neurological disorders. Patients undergo determination of candidacy, then surgery if appropriate and patient is willing. Both patients and families generally participate in the programming process (38). In DBS candidacy determinations for movement disorders, all active psychiatric comorbidities must be identified and managed prior to surgery (38, 39). Presurgical evaluation often involves assessment of family and/or other social support (40). Severe SUD are associated with numerous psychiatric conditions, including personality disorders (41), and many individuals with severe SUD have disrupted family connections, socioeconomic challenges, and criminal justice involvement (42). Thus, while many patients with Parkinson’s disease recover post-operatively at home with supportive family members (e.g., providing a stable recovery environment), individuals with SUD may have limited support. DBS programming typically begins several weeks after surgery to allow for resolution of peri-lead post-operative edema which can temporarily suppress symptoms and confound determination of stimulation effect. Patients with severe, treatment-refractory SUD are at risk for relapse during this post-operative period. Thus, trials of DBS for individuals with SUD generally provide residential care following surgery, which adds logistical challenges and costs. These patients may also benefit from additional support (e.g., case management) upon discharge from residential care. In addition, SUDs can be viewed in part as subverting motivational systems and prioritizing drug reinforcement (e.g., pursuit of the drug to the detriment of roles/positive relationships), leading to fluctuations in level of treatment motivation across time. Thus, studies of DBS for SUD are faced with a conundrum: recruit individuals with severe, treatment-refractory SUD, but prioritize those who have more treatment motivation and family support and hence may be more likely to comply with long-term study participation requirements. Such individuals, however, represent a small subsample of those with SUD.

The potential for harm from DBS must be considered in patient selection. Prior work has reported suicidal ideation and completed suicides during DBS treatment for other conditions (43), leading some to conclude that those at high risk for suicide should be excluded from DBS consideration (44), though this has been debated in depression trials (45). Those with severe SUD may also have co-morbid serious medical concerns which must be carefully assessed in relation to DBS risks. Another concern is the risk of infection due to bacterial colonization of the DBS hardware (46). Should people who inject drugs be excluded from enrollment in DBS studies given potential for increased infection risk? Excluding these individuals may exclude those with the greatest need. SUDs in the US are commonly associated with criminal justice involvement/incarceration (47). Given that studies of DBS involve a small number of individuals enrolled over long periods, what happens if a patient enrolled in a DBS trial becomes incarcerated?

### Trial design issues

Although early open label studies have suggested long-term improvement in SUD phenotypes (i.e., long-term remission), there are multiple design choices which may impact confidence in trial results, including target selection, surgical approach, approach to programming, expected time courses, and blinding.

Which target? The neurobiological understanding of SUD has grown dramatically from a focus mainly on reward-related processes (the dopamine hypothesis) to including network based models, and including a broad number of brain regions and circuits (48, 49). Thus, target selection for the treatment of a specific SUD is complex. Animal models have been used to test a broad range of DBS targets including the subthalamic nucleus, substantia nigra, amygdala, anterior cingulate, insula, hippocampus, infralimbic and prelimbic cortices, along with NAc (50–55). Cortical regions (e.g., dorsolateral prefrontal cortex, ventromedial prefrontal cortex, and anterior cingulate) are generally accessible to TMS, and studies of DBS generally focus on subcortical regions. To our knowledge, DBS in SUD, including early case reports showing substance use outcomes in patients treated for other conditions (25, 26, 30), has generally focused on NAc, anterior limb of the internal capsule, bed nucleus of the stria terminalis, and ventral capsule-ventral striatum region (a broader region which includes NAc) (12). However, a trial focusing on the limbic pallidum for alcohol use disorder was funded in 2022 (AA030505). Current DBS lead technology is not ideally suited to differentially stimulate intimately adjacent structures (e.g., shell vs. core of NAc). In such instances, stimulation might inadvertently result in opposing effects—e.g., lateral versus medial NAc shell (56) or core versus shell (50), or vary depending on white matter fiber connections to cortical regions (57). The large number of candidate targets speaks to limitations in our current understanding of the fundamental pathophysiology and neural circuitry of SUD, but also highlights the possibility that thoughtful target selection informed by our current neurobiological models, may further inform our understanding of these disorders.

#### Surgical planning

The use of 3T MRI-guidance (or CT-guided with MRI fusion) for stereotactic procedures allows accurate targeting of many brain structures. Individual anatomy may require adjustments to trajectory—for instance, the need to avoid cortical veins, which are highly variable between individuals, or the targeting of white matter tracts, if indicated, which also vary between individuals. Significant brain-shift from opening the dura and subsequent air entry is a potential targeting confound when using preoperative image guidance. Standard surgical techniques can be used to reduce the likelihood of significant brain shift—such as positioning the patient with minimal head-of-bed elevation, and the use of intraoperative MRI to account for any brain shift during the surgical procedure is also an option.

#### Programming plan

In Parkinson’s disease, tremor, rigidity, and bradykinesia provide quantifiable symptoms against which to set stimulation parameters and select active contacts. Although a half-smile may be observed during DBS programming for obsessive compulsive disorder (58), stimulation settings for psychiatric conditions generally rely on subjective self-report (e.g., patient self-rating of mood, anxiety, energy, and side effects) to identify the optimal stimulation settings. It remains unclear whether self-report represents a reliable and valid measure for guiding stimulation parameter settings in SUD. The subjectivity of response and complexity of the DBS programming parameter space (**Figure 1**) presents significant challenges (59). Thus, a pressing goal in DBS for the treatment of psychiatric conditions is to identify objective measures for optimizing stimulation parameters (60), such as using symptom-relevant features of the local field potential (LFP) spectrum (61) and imaging NAc during cue craving, as has been done for food craving (62). Many of the published case reports on DBS for SUD include the final stimulation parameter settings (12), but do not describe the approach to optimizing these parameters. Post-operative brain imaging can be used to inform contact selection for programming by examining location of individual contacts and through estimation of volume of tissue activation [i.e., shaping the stimulation field based on local anatomy (63)]. Bach and colleagues (13) describe a proscribed stimulation protocol for parameter selection in their recent RCT, which would be easily reproducible in clinical practice, analogous to the careful dosing guidelines used in medication trials.

#### Time course to expected response

A challenge in DBS trial design for SUD is predicting time to clinical response. For depression, treatment response may take a minimum of 6 months or as long as 1-2 years, with increasing response rates generally observed with longer follow-up (43, 64). *Post-hoc* assessment of the BROADEN Trial (subgenual cingulate white matter stimulation for treatment-refractory depression) data has suggested that prolonged time to response may have played a role in the interim futility analyses that halted the trial. This experience has underscored the importance of designing trials of DBS for SUD to capture long-term effects (65). Furthermore, the time necessary for washout of the effects of stimulation is unclear, which creates difficulties in designing cross-over studies (i.e., it is unclear how long is needed for a subject’s symptoms to return to baseline after discontinuation of stimulation).

For SUD, response rates have been reported to occur both rapidly/immediately following onset of stimulation (29) and as late as 10 months after beginning stimulation (30). The only double-blind RCT of DBS for SUD published to date, used a 6-month follow up period, with one group off and one on stimulation, followed by open-label stimulation for 12 months. Symptom amelioration, on average, was seen within the first two weeks of stimulation (13). As noted above, residential treatment may be used during the post-surgical recovery period, and may impact relapse rates at 3 months, and even 1 year, after discharge (66); effects may be confounded by adjunctive treatments in open-label designs. Finally, microlesion effects (i.e., post-operative edema in the targeted brain structure) have been previously reported up to 2-3 months following surgery (67).

#### Trial design-placebo and blinding

As with pharmaceutical interventional studies for psychiatric conditions, blinding provides protection against bias in neuromodulation studies. DBS case reports and case series can be criticized for lacking both blinding and sham stimulation. There are, however, challenges to blinding and design of sham-control arms in DBS studies (**Figure 1**). Because DBS surgery and residential substance treatment is expensive, and the follow-up period is long, studies will likely only enroll a small number of subjects. Crossover designs using the same participant on- and off-stimulation may enhance power and account for ordering effects.

### Ethical issues

DBS for treatment of patients with SUD raises several ethical considerations including, but not limited to, considerations around informed consent and trial completion.

#### Informed consent

Patients with SUD are not *de facto* challenged from a decision-making vantage and are capable of providing informed consent for study participation, though such consent *cannot* be given in the context of acute intoxication or withdrawal. When selecting patients who are severely affected and treatment-refractory for a novel intervention, there is always the potential for misunderstanding the likelihood of treatment effect and the experimental nature of the study. A patient advocate can provide an additional and important voice during these meetings. Managing patient expectations is an important part of the informed consent process and utilization of visual aids to help potential participants understand the surgical procedures is paramount. Our study employs a Multi-disciplinary Ethics Board, chaired by a bioethicist, and includes our patient advocate, and outside representatives with expertise in Addiction Psychiatry, Neurology, and Neurosurgery. This board reviews all potential participants recommended by the study team and prevents surgery with a single “No” vote.

#### Battery life, concluding the trial, costs

DBS leads and extension cables are permanently implanted devices while the IPG has a finite lifespan, the duration of which is dependent on stimulation parameters and whether or not a rechargeable IPG is used. Currently available IPGs—including rechargeable IPGs— require periodic and repeated replacement over the life of the patient. The life of the IPG is related to the therapeutic stimulation parameters selected, with certain configurations (e.g., higher mA) causing faster battery depletion. Battery life can range from 7 months to several years, requiring plans for possible IPG replacement surgery over the study duration. Rechargeable batteries can increase time until battery replacement, but recharging may pose compliance issues for some patients with SUD. Sensing and storage of LFP signals will also shorten battery life. At the conclusion of study participation for a medication trial, the subject no longer uses the study medication. At the conclusion of a DBS trial, if a participant is doing well, who will cover the cost of IPG replacement surgery or ongoing care? Should enrollment in a longer-term open-label follow-up study be offered? Who will cover costs if explantation is required? Such questions have been considered previously (68), but we are unaware of any clear guidance and each research team must carefully consider such questions at the outset of the trial and include relevant information in the informed consent process for study participation.

## Discussion

Studies of DBS for treatment-refractory SUD are currently underway in the US, Europe, and Asia, and offer hope for more effective treatments and enhanced understanding of the neurobiological underpinnings of SUD. Early results of single case reports and open-label trials have been promising, with some individuals experiencing sustained remission (12). However, such trial results come with many important considerations and concerns. From our experience in planning and implementing one such study, we present five recommendations to guide future DBS trials for SUD.

(1) Case reports and small, open-label studies have now been published suggesting that DBS of the NAc may provide benefit to individuals with different SUDs. Future studies must implement more rigorous trial design to control for placebo effects, which may be nontrivial with surgical intervention (69) and in addiction treatment (70). Because of costs and the usually extensive requirements for participant selection, sample sizes will generally remain small, but additional open-label studies will be inadequate to convincingly demonstrate efficacy. Multi-site studies may be required, along with utilization of designs which maximize power with modest sample sizes (e.g., cross-over design), to push the field forward. The field should also focus on development of standard approaches to sham programming that maintains the blind.
(2) **Table 1** reviews the available literature on adverse events in DBS for SUD trials. It is important to note that the available data do not raise new safety concerns for DBS in this population. However, many prior papers do not provide details on how adverse events were monitored and because of an open trial design, cannot comment on rates of adverse events in sham vs. active stimulation. Adverse events should be systematically assessed and reported in all trials to better understand risks in this population. Careful assessment of adverse events will also help inform future potential participants and investigators regarding the risk-to-benefit ratio.
(3) Prior studies often do not carefully explain approaches to stimulation parameter setting or subsequent optimization. Reproducibility requires careful protocolization of stimulation parameters and documentation of those approaches. Ideally, future studies will collect objective markers (e.g., MRI, LFP) which may aid in the development of objective measures to guide programming.
(4) Unlike medication trials, DBS trials involve surgical implantation of hardware. Because of this, research teams should work to carefully anticipate long-term patient needs and provide information to potential participants during the informed consent process about what can, and cannot, be provided throughout the study (e.g., explantation, battery replacement, etc.).
(5) Ethical issues in DBS for SUD (from patient selection and informed consent to conduct and conclusion of the trial) must be delineated and managed in protocol development.

Future Directions: DBS, in relation to other neuromodulation techniques, may offer some advantages which should be pursued. For example, DBS with its ability to provide long-term stimulation to deep structures likely enhances durability and precision of treatment response (e.g., relative to some noninvasive stimulation approaches) and this should be investigated. Advances in the measurement of symptom-relevant LFP-derived biomarker may also inform our understanding of the neurobiology of addiction and may open pathways to develop closed loop approaches (e.g., IPG senses LFP changes signifying craving and provides stimulation). Thus, while increasing rigor is needed to define treatment effects, these technologies may offer ways to advance our fundamental understanding of addiction-related process.

## Data Availability

The original contributions presented in the study are included in the article/supplementary material. Further inquiries can be directed to the corresponding author.
