# Corrigendum: Deep brain stimulation for the treatment of substance use disorders: a promising approach requiring caution

**DOI:** 10.3389/fpsyt.2025.1559114

**Published:** 2025-05-22

**Authors:** Joseph T. Sakai, Jody Tanabe, Sharonya Battula, Morgan Zipperly, Susan K. Mikulich-Gilbertson, Drew S. Kern, John A. Thompson, Steven Ojemann, Kristen Raymond, Pamela David Gerecht, Katrina Foster, Aviva Abosch

**Affiliations:** ^1^ Department of Psychiatry, University of Colorado School of Medicine, Aurora, CO, United States; ^2^ Department of Radiology, University of Colorado School of Medicine, Aurora, CO, United States; ^3^ Department of Neurology, University of Colorado School of Medicine, Aurora, CO, United States; ^4^ Department of Neurosurgery, University of Colorado School of Medicine, Aurora, CO, United States; ^5^ National Institute on Drug Abuse, Bethesda, MD, United States; ^6^ Department of Neurosurgery, University of Nebraska Medical Center, Omaha, NE, United States

**Keywords:** substance use disorder, addiction, brain stimulation, neuromodulation, invasive, craving

In the published article, there was an error in the author list. Steve Ojemann was erroneously excluded.

In the published article, there was an error in the **Author Contributions** section. Steve Ojemann (SO), was erroneously excluded. The corrected statement appears below:

“JS: Conceptualization, Funding acquisition, Writing – original draft, Writing – review & editing. JT: Conceptualization, Funding acquisition, Writing – review & editing. SB: Writing – review & editing. MZ: Writing – review & editing. SM-G: Writing – review & editing. DK: Writing – review & editing. JAT: Writing – review & editing. SO: Writing – review & editing. KR: Writing – review & editing. PG: Writing – review & editing. KF: Writing – review & editing. AA: Conceptualization, Funding acquisition, Writing – review & editing.”

The authors apologize for these errors and state that this does not change the scientific conclusions of the article in any way. The original article has been updated.

